# Plasma and CSF Neurofilament Light Chain in Amyotrophic Lateral Sclerosis: A Cross-Sectional and Longitudinal Study

**DOI:** 10.3389/fnagi.2021.753242

**Published:** 2021-10-22

**Authors:** Veria Vacchiano, Andrea Mastrangelo, Corrado Zenesini, Marco Masullo, Corinne Quadalti, Patrizia Avoni, Barbara Polischi, Arianna Cherici, Sabina Capellari, Fabrizio Salvi, Rocco Liguori, Piero Parchi

**Affiliations:** ^1^Department of Biomedical and NeuroMotor Sciences (DIBINEM), University of Bologna, Bologna, Italy; ^2^IRCCS Istituto delle Scienze Neurologiche di Bologna, Bologna, Italy; ^3^Department of Experimental Diagnostic and Specialty Medicine (DIMES), University of Bologna, Bologna, Italy

**Keywords:** neurofilament light chain, amyotrophic lateral sclerosis, diagnosis, prognosis, longitudinal, Simoa, biofluid, biomarker

## Abstract

**Background:** Neurofilament light chain (NfL) is a validated biofluid marker of neuroaxonal damage with great potential for monitoring patients with neurodegenerative diseases. We aimed to further validate the clinical utility of plasma (p) vs. CSF (c) NfL for distinguishing patients with Amyotrophic Lateral Sclerosis (ALS) from ALS mimics. We also assessed the association of biomarker values with clinical variables and survival and established the longitudinal changes of pNfL during the disease course.

**Methods:** We studied 231 prospectively enrolled patients with suspected ALS who underwent a standardized protocol including neurological examination, electromyography, brain MRI, and lumbar puncture. Patients who received an alternative clinical diagnosis were considered ALS mimics. We classified the patients based on the disease progression rate (DPR) into fast (DPR > 1), intermediate (DPR 0.5–1), and slow progressors (DPR < 0.5). All patients were screened for the most frequent ALS-associated genes. Plasma and CSF samples were retrospectively analyzed; NfL concentrations were measured with the SIMOA platform using a commercial kit.

**Results:** ALS patients (*n* = 171) showed significantly higher pNfL (*p* < 0.0001) and cNfL (*p* < 0.0001) values compared to ALS mimics (*n* = 60). Both cNfL and pNfL demonstrated a good diagnostic value in discriminating the two groups, although cNfL performed slightly better (cNfL: AUC 0.924 ± 0.022, sensitivity 86.8%, specificity 92.4; pNfL: AUC 0.873 ± 0.036, sensitivity 84.7%, specificity 83.3%). Fast progressors showed higher cNfL and pNfL as compared to intermediate (*p* = 0.026 and *p* = 0.001) and slow progressors (both *p* < 0.001). Accordingly, ALS patients with higher baseline cNfL and pNfL levels had a shorter survival (highest tertile of cNfL vs. lowest tertile, HR 4.58, *p* = 0.005; highest tertile of pNfL vs. lowest tertile, HR 2.59, *p* = 0.015). Moreover, there were positive associations between cNfL and pNfL levels and the number of body regions displaying UMN signs (rho = 0.325, *p* < 0.0001; rho = 0.308, *p* = 0.001). Finally, longitudinal analyses in 57 patients showed stable levels of pNfL during the disease course.

**Conclusion:** Both cNfL and pNfL have excellent diagnostic and prognostic performance for symptomatic patients with ALS. The stable longitudinal trajectory of pNfL supports its use as a marker of drug effect in clinical trials.

## Introduction

Amyotrophic lateral sclerosis (ALS) is a heterogeneous neurodegenerative disorder affecting both the upper (UMN) and the lower motor neurons (LMN). It generally causes progressive and diffuse muscular paralysis and eventually affects the nutritional and respiratory functions leading to death. The diagnosis of ALS currently relies on the demonstration of clinical and electrophysiological signs of damage of motor neurons at both levels and the exclusion of ALS mimics (Brooks et al., 2000; de Carvalho et al., 2008). Whereas ALS in its “classical” phenotype rarely represents a diagnostic challenge for an experienced neurologist, ALS variants with prevalent UMN or LMN involvement may present with subtle, slowly progressive clinical signs and lead to misdiagnosis with alternative disorders (Swinnen and Robberecht, 2014). Thus, the definition of CSF and blood disease biomarkers is of great relevance to improve the diagnostic accuracy and the prognostic assessment of patients. Moreover, as potential disease-modifying approaches for neurodegenerative disorders are emerging, there is an urgent need for biomarkers to monitor the therapeutic effect during the disease course.

Among several candidates, neurofilament light chain (NfL), a validated marker of neuroaxonal damage that can be reliably measured in both CSF (cNfL) and plasma (pNfL) (Gray et al., 2020), showed the best performance in distinguishing patients with ALS from patients with ALS mimics (Steinacker et al., 2016; Poesen et al., 2017; Feneberg et al., 2018;Gille et al., 2019; Abu-Rumeileh et al., 2020; Ashton et al., 2021).

Moreover, several authors highlighted the potential role of cNfL and pNfL as robust prognostic biomarkers, given the significant associations between the disease progression rate (DPR) and survival and the basal biomarkers values (Lu et al., 2015; Gaiani et al., 2017; Poesen et al., 2017; Steinacker et al., 2017; Feneberg et al., 2018; Benatar et al., 2020; Thouvenot et al., 2020). Finally, a few preliminary longitudinal studies suggested that pNfL levels remain stable in the disease course (Lu et al., 2015; Skillbäck et al., 2017; Verde et al., 2019; Benatar et al., 2020), making this novel biomarker a potential candidate for the monitoring of future therapeutic approaches in ALS.

In this study, we aimed to further explore the value of cNfL vs. pNfL in distinguishing patients with ALS and ALS mimics in one of the largest cohorts studied to date. Furthermore, we assessed the association of both biomarkers with clinical variables and with survival. Finally, we sought to describe the longitudinal behavior of pNfL, analyzing the biomarker values at different disease stages in a significant group of patients.

## Materials and Methods

### Ethical Approval

The study was conducted according to the revised Declaration of Helsinki and Good Clinical Practice guidelines. Written informed consent was given by study participants. The study was approved by the ethics committee of “Area Vasta Emilia Centro.”

### Inclusion Criteria and Clinical Assessment

We studied 171 ALS patients and 60 patients with an alternative clinical diagnosis (ALS mimics group) evaluated at the Institute of Neurological Sciences of Bologna (ISNB) between September 2014 and June 2021. We also analyzed blood and CSF samples from 57 non-neurodegenerative controls, namely 30 blood samples from healthy subjects and 27 CSF samples from patients lacking any clinical or neuroradiological evidence of central nervous system (CNS) disease.

Patients with suspected ALS were prospectively enrolled, and underwent a standardized protocol including neurological examination, electromyography (EMG), lumbar puncture and ancillary exams to exclude an alternative clinical diagnosis. We included in the ALS group patients who received a diagnosis of ALS according to the Revised El Escorial criteria at baseline or during follow-up (Brooks et al., 2000), with available clinical data and at least one between CSF and plasma samples at baseline. Patients evaluated for ALS who received an alternative clinical diagnosis during the diagnostic work-up and/or follow-up and with at least one biofluid available were included in the ALS mimics group. For ALS patients the following clinical data were collected at the time of diagnosis (baseline visit): Age at onset, sex, disease duration (time elapsed between the first referred symptom and sampling), type of onset [bulbar, spinal, pseudopolyneuritic or pyramidal (Swinnen and Robberecht, 2014)], clinical phenotype [classical, bulbar, predominant upper motor neuron (PUMN), predominant lower motor neuron (PLMN) (Chiò et al., 2011; Al-Chalabi et al., 2016)], ALS Functional Rating Scale-revised (ALSFRS-R) score, forced vital capacity (FVC) expressed as a percentage of predicted volume, and body mass index (BMI). Patients were classified according to the Revised El Escorial criteria in 31 definite ALS, 69 probable ALS, 31 probable laboratory-supported ALS and 40 possible ALS (Brooks et al., 2000), and staged in agreement with King’s clinical staging system (Roche et al., 2012). All patients underwent genetic screening for the most frequent ALS genes (i.e., *SOD1, FUS*, *TARDBP*, and the repeats expansion of the *C9Orf72* gene) (Bartoletti-Stella et al., 2021). The degree of the UMN involvement was defined as the number of regions (bulbar, cervical and lumbosacral region) showing UMN signs at clinical examination, while for the extent of the LMN involvement both clinical and EMG assessment were considered, as stated by the Awaji criteria (de Carvalho et al., 2008). The DPR at the baseline visit was calculated as follows: (48-ALSFRS-R score at the time of sampling)/months elapsed between disease onset and sampling (Lu et al., 2015), and patients were accordingly divided into slow (DPR < 0.5), intermediate (DPR 0–5–1) and fast progressors (DPR > 1), as previously described (Lu et al., 2015). Moreover, the Medical Research Council (MRC) scale of 0–5 (calculated as the sum of 10 muscles for each side score/20; score 0–5 points) was provided for each patient at the time of clinical evaluation.

A subgroup of ALS patients underwent the Edinburgh Cognitive and Behavioral ALS Screen (ECAS) (Abrahams et al., 2014; Siciliano et al., 2017) to investigate the presence of cognitive impairment up to a full-blown frontotemporal dementia (FTD).

Baseline CSF and plasma samples were used for a cross-sectional study of NfL levels.

Fifty-seven of the 171 ALS patients had plasma samples available from two or more visits. Longitudinal plasma samples were obtained during multidisciplinary follow-up visits from ALS patients who accepted to donate further blood samples after baseline sampling. No selection criteria were applied to identify these patients. In details, 24 patients were sampled twice, 20 patients had three plasma samples, 11 patients were sampled four times and for two subjects we had five samples available. Patients were repeatedly sampled at non-standardized time points, with a median follow-up period of 12 months (IQR 8–26). We observed 55 patients for more than 3 months, 50 subjects for at least 6 months, 32 and 17 patients for at least 12 and 24 months, respectively. The most extended follow-up duration was 55 months (two patients). For patients with more than one sampling, we calculated the longitudinal disease progression rate (l-DPR), as the change in the ALSFRS-R between the last and the baseline visits divided by the number of months between the visits (Vu et al., 2020). Accordingly, ALS patients were further classified into fast progressors (l-DPR > 1), intermediate progressors (l-DPR 0.5–1), and slow progressors (l-DPR < 0.5).

### CSF and Plasma Analyses

EDTA plasma samples were collected, aliquoted, and stored at −80°C according to standard procedures. CSF samples were obtained by LP following a standard procedure, centrifuged in case of blood contamination, divided into aliquots, and stored in polypropylene tubes at −80°C until analysis.

Both cNfL and pNfL concentrations, in the entire sample cohort, were determined with the Single molecule array (Simoa) technology on a Simoa SR-X instrument (Quanterix, Billerica, MA, United States) using the commercially available NF-light advantage kit (Quanterix). The mean intra- and inter-assay coefficients of variation (CVs) were below 15% for both cNfL and pNfL.

### Genetic Analyses

Molecular genetic analyses were performed as previously described (Bartoletti-Stella et al., 2021). Briefly, genomic DNA (gDNA) was extracted from peripheral blood by standard procedures (Giannoccaro et al., 2017). gDNA was quantified using the Quantus Fluorometer (Promega) with QuantiFluor double stranded DNA system (Promega). Patients were screened for mutations in ALS major genes: *SOD1* (all exons), *FUS* (exons 6 and 15), *TARDBP* (exons 2, 3, and 5) genes and for pathogenic repeat expansion (RE) in the *C9orf72* gene as previously reported (Bartoletti-Stella et al., 2021).

### Statistical Analyses

Statistical analyses were performed using IBM SPSS Statistics version 21 (IBM, Armonk, NY, United States), Stata SE version 14.2 (StataCorp LLC, College Station, TX, United States) and GraphPad Prism 7 (GraphPad Software, La Jolla, CA, United States) software.

Continuous variables were presented as mean and Standard Deviation (SD) or median and interquartile range (IQR), categorical variables were presented as absolute number (n) and relative frequency (%). For continuous variables, based on the data distribution, the Mann-Whitney U test or the Student *t*-test were adopted to evaluate the differences between the groups, while the Kruskal-Wallis test (followed by Dunn-Bonferroni *post hoc* test) or the one-way analysis of variance (ANOVA) (followed by Tukey’s *post hoc* test) were used for multiple group comparisons. Chi-Square test was applied for categorical variables. Biomarker values were transformed into a logarithmic scale to obtain a normal data distribution.

For the analysis of diagnostic value, receiver operating characteristic (ROC) analyses were performed to establish the accuracy in the distinction between ALS and ALS mimics, as well as the sensitivity and specificity of biomarkers. The optimal cut-off value for each biomarker was calculated using the maximed Youden Index. A subgroup analysis was also carried out according to patients’ median age (≤55 vs. > 55 years) and sex (female vs. male). De Long test was used to compare the areas under the curve of pNfL and cNfL in the whole groups and between subgroups.

For the cross-sectional analysis, Spearman’s rho coefficient was used to test the correlation between cNfL and pNfL levels and clinical variables. Moreover, the association between biofluids NfL and the degree of UMN and/or LMN involvement was analyzed using univariate and multivariate linear regression models with the log-transformed biomarker values (cNfL and pNfL) as dependent variables and the extent of: (1) UMN involvement, (2) LMN involvement, (3) UMN and LMN involvement as independent variables. In the multivariable models we adjusted for age at sampling, sex, genetic status, basal ALSFRS-R score, DPR, MRC and King’s scores. The results are presented as β coefficients and 95% confidence intervals (95% CI).

For the prognostic analysis the cumulative time-dependent probability of death was calculated by the Kaplan-Meier estimate. The time of entry into the analysis was the date of the first sampling (at baseline), and the time of the endpoint was the date of death/tracheostomy or the date of the last follow-up information, whichever came first. We performed univariate and multivariate Cox regression models to study the association between time to death/tracheostomy and prognostic factors in ALS. The multivariate Cox regression analysis was adjusted for age at baseline, sex, baseline ALSFRS-R score, genetic status, DPR, MRC and King’ scores. The results are presented as Hazard Ratios (HR) and 95% CI. The assumption of proportional hazard was assessed by Schoenfeld residuals. Differences were considered significant at *p* < 0.05.

For the longitudinal analysis, a linear mixed effect modeling analysis with random slope and random intercept was performed to evaluate the rate of change over the time of both cNfL and pNfL in the ALS patients stratified into fast, intermediate and slow progressors, as previously described (Vu et al., 2020). The results are presented as β coefficients and 95% CI.

## Results

### Demographic Values, Distribution and Diagnostic Performance of Plasma Neurofilament Light Chain and Cerebrospinal Fluid Neurofilament Light Chain

Demographic and clinical features of the study population are detailed in Tables 1, 2 and Supplementary Table 1.

**TABLE 1:** Demographic and clinical features of the study population.

ALS patients—clinical characteristics	N (tot. 171)	%
*Sex*	68 (F)	39.8
*Type of onset*		
Bulbar	42	24.6
Spinal	113	66.1
Pseudopolyneuritic	9	5.3
Pyramidal	7	4.1
Deceased/with tracheostomy	72	42.1
*Genetic screening*	(N tot. 167)	
*C9Orf72* RE carriers	18	10.8
*SOD1* mutation carriers	7	4.2
*FUS* mutation carriers	1	0.6
*TARDBP* mutation carriers	2	1.2
*FTD status*	21	12.3
		Median (IQR)
*Age at first sampling (y)*		65 (56–74)
*DD from first symptom to sampling (m)*		16 (9–27)
*ALSFRS-R score*		41 (34.5–44)
*MRC score*		4.6 (4.1–4.8)
*FVC*		90 (70–106)
*Biomarker values*		Median (IQR)
cNfL	114	6543 (3697–12719)
pNfL	170	73.0 (45.9–114.2)
**ALS mimics group**	**N (tot. 60)**	**%**
*Sex*	24 (F)	40
*Age at first sampling (y)*		Median (IQR)
		65 (56.3–71.8)
*Biomarker values*		Median (IQR)
cNfL	53	1140 (589.5–1937)
pNfL	30	22.5 (11.4–28)
**Clinical and healthy controls**	N (tot. 57)*	**%**
*Sex*	26 (F)	45.6
*Age at sampling (y)*		Median (IQR)
		63 (56.5–69)
*Biomarker values*		Median (IQR)
cNfL	27	682.3 (498.7–934.3)
pNfL	30	9.4 (6.8–15.5)

*Clinical and healthy controls used for cNfL and pNfL analysis are presented as a single group because they did not differ significantly in median age and sex distribution. Biomarker values are in pg/ml. Key: ALS, amyotrophic lateral sclerosis; ALSFRS-R, Revised Amyotrophic Lateral Sclerosis Functional Rating; cNfL, cerebrospinal fluid neurofilament light chain; DD, disease duration; FVC, forced vital capacity; FTD, frontotemporal dementia; IQR, interquartile range; m, months; MRC, Medical Research Council; PLMN, predominant lower motor neuron; pNfL, plasma neurofilament light chain; PUMN, predominant upper motor neuron; RE, repeats expansion; y, years.

**TABLE 2:** Diagnostic categories in the ALS mimics group.

ALS mimic diagnoses	60
Hereditary or idiopathic spastic paraplegia	12
Chronic inflammatory demyelinating polyneuropathy	5
Polyneuropathy	6
Myelopathy/myelitis	3
Multineuropathy	3
Spinal muscular atrophy 3	2
Myopathy/myositis	4
Cramp-fasciculation syndrome	1
Spinocerebellar ataxia	1
Focal amyotrophy	2
Amyloidosis	1
Myasthenia gravis	3
Post-polio syndrome	1
Caspr2 antibody-associated disease	1
Anti-IgLON5 disease	1
Meningioma	1
Hydrocephalus	1
PSP-PLS	1
Atypical parkinsonism	2
Alexander’s disease	1
Lumbar spinal stenosis	1
Unclassified	7

*Key: ALS, amyotrophic lateral sclerosis; PLS, primary lateral sclerosis; PSP, progressive supranuclear palsy.*

Age at baseline and sex distribution were not significantly different among the three diagnostic groups (age, *p* = 0.575; sex, *p* = 0.728). No effect of sex and age on cNfL and pNfL values was detected in the ALS group, while there was a moderate effect of age on pNfL and cNfL levels in both the ALS mimics (age vs. pNfL: rho = 0.546, *p* < 0.001; age vs. cNfL: rho = 0.536, *p* < 0.001) and the control groups (age vs. pNfL: rho = 0.691, *p* < 0.001; age vs. cNfL: 0.451, *p* = 0.018).

**FIGURE 1:**
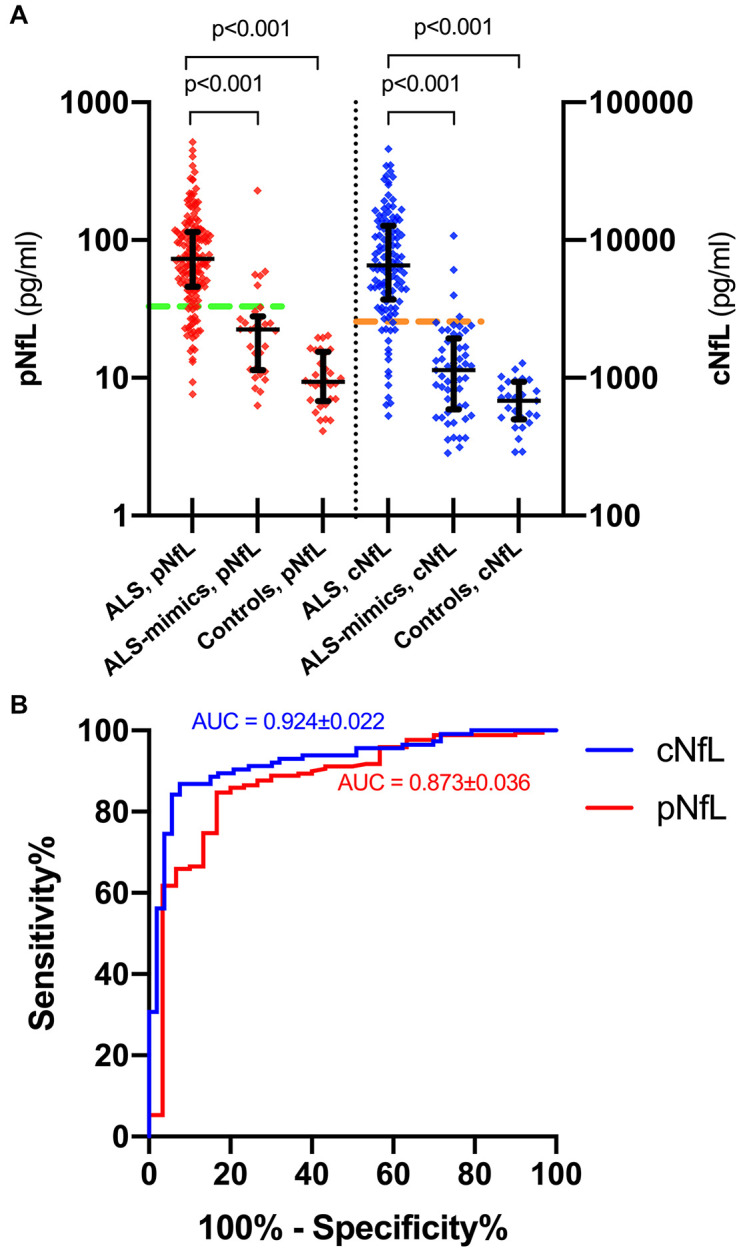
pNfL and cNfL levels in the diagnostic groups and ROC curves for pNfL and cNfL. Both cNfL and pNfL demonstrate high diagnostic value in the distinction between ALS and ALS mimics. **(A)** pNfL and cNfL levels in ALS patients, ALS-mimics and control groups. Thick lines represent medians and interquartile ranges. Biomarker values are expressed in the logarithmic scale. Dotted horizontal lines indicate the optimal cut-off values for pNfL (green) and cNfL (orange) in the distinction between ALS and ALS-mimics patients, as calculated through the maximized Youden Index. Only *p*-values of significative comparisons are shown (Kruskal-Wallis followed by Dunn-Bonferroni *post hoc* test). **(B)** ROC curves for pNfL (red) and cNfL (blue) in the comparison between ALS patients and ALS-mimics. Key: cNfL, cerebrospinal fluid neurofilament light chain; pNfL, plasma neurofilament light chain.

ALS patients showed significantly higher pNfL (*p* < 0.0001) and cNfL (*p* < 0.0001) values compared to subjects belonging to the ALS mimics and control groups (Figure 1A). When evaluating the ROC curves, cNfL yielded a higher diagnostic value than pNfL (*p* = 0.043) in discriminating patients with ALS and subjects with an alternative ALS-mimicking disease (cNfL: AUC 0.924 ± 0.022, sensitivity 86.8%, specificity 92.4, cut-off 2,517 pg/ml; pNfL: AUC 0.873 ± 0.036, sensitivity 84.7%, specificity 83.3%. cut-off 32.7 pg/ml) (Figure 1B). After patient stratification, we found no significant influence of age (*p* = 0.149) and sex (*p* = 0.644) on the diagnostic performance of cNfL. Age but not sex (*p* = 0.981) slightly influenced the diagnostic accuracy of pNfL, although the effect did not reach statistical significance (≤ 55 years: AUC 0.939 ± 0.026 vs. > 55 years: AUC 0.804 ± 0.067; *p* = 0.062). Finally, the diagnostic accuracy of pNfL almost reached that of cNfL (AUC 0.906 ± 0.026, sensitivity 84.7%, specificity 86.4%, cut-off 32.7 pg/ml), when we limited the analysis to the subjects with alternative diseases only involving the CNS.

### Association Between Cerebrospinal Fluid Neurofilament Light Chain, Plasma Neurofilament Light Chain and Clinical Variables

cNfL and pNfL values strongly correlated at baseline (Spearman’s rho = 0.836, *p* < 0.0001).

When evaluating the associations between biofluid biomarkers and measures of ALS severity, we found a marked association between both cNfL and pNfL concentrations and DPR (rho = 0.493, *p* < 0.0001; rho = 0.525, *p* < 0.0001, respectively), and a weaker association of NfL values in both biofluids with the MRC score (rho = 0.231, *p* = 0.014; rho = 0.248, *p* = 0.002), FVC (rho = 0.363, *p* = 0.003; rho = 0.276, *p* = 0.001), and ALSFRS-R (rho = 0.206, *p* = 0.023; rho = 0.217, *p* = 0.006) values. cNfL levels were also weakly correlated with the King’s stage (rho = 0.249, *p* = 0.008).

Moreover, fast progressors (i.e., ALS patients with DPR > 1) showed higher cNfL and pNfL compared to intermediate (*p* = 0.026 and *p* = 0.001) and slow progressors (*p* < 0.001).

In contrast, there was no significant association between pNfL/cNfL and ECAS (total, ALS-specific and ALS non- specific scores) and BMI, and between pNfL and King’s stage. cNfL levels significantly differed across onset types (*p* = 0.011), and *post hoc* analysis revealed significantly higher levels in patients with bulbar than in those with spinal onset (*p* = 0.038). We found no significant differences across ALS variants, FTD, or genetic status, although cNfL resulted higher in ALS-FTD patients than in pure ALS (8637.2, IQR 6331.9-13979.9 vs. 6155.7, IQR 3231.4-12011, *p* = 0.093) and in *C9Orf72* RE carriers (*p* = 0.14) (Table 3).

**TABLE 3:** pNfL and cNfL levels according to the accuracy level of the categories of the Revised El Escorial diagnostic criteria and to genetic status (i.e., wild type vs. ALS gene mutations).

Revised El *Escorial criteria*	N	pNfL Median (IQR)	N	cNfL Median (IQR)
Possible ALS	38	64.9 (27.6–101.3)	25	4536 (2232–8853)
Probable laboratory-supported ALS	31	45.8 (31.4–70.7)	24	5100 (3145.2–7760)
Probable ALS	68	86.1 (57.7–127.2)	45	7572 (4770–15569)
Definite ALS	31	100.5 (58.8–135.5)	19	10892.4 (6156–14629)
*Genetic status*	N	pNfL Median (IQR)	N	cNfL Median (IQR)
Wild-type	137	73.5 (43.9–113.7)	98	6317 (3574–13476)
*SOD1*	6	36.0 (14.0–59.4)	2	2252; 4536
*TARDBP*	2	32.8; 51.8	1	3018.5
*FUS*	1	37.2	0	NA
*C9Orf72*	18	107.1 (64.5–125.3)	11	10796 (7950–12031)

*Biomarker values are expressed in pg/ml. Key: ALS, amyotrophic lateral sclerosis; cNfL, cerebrospinal neurofilament light chain; IQR, interquartile range; N, number; NA, not available; pNfL, plasma neurofilament light chain.*

pNfL levels did not significantly differ among ALS phenotypes and type of onset but were slightly increased in FTD-ALS patients compared to those with ALS alone, with a trend of significance (110.8, IQR 55.5-165 vs. 70.7, IQR 43.4-109.5, *p* = 0.054). Moreover, pNfL values were significantly higher in *C9Orf72* RE expansion carriers than in the other patients (*p* = 0.010) (Table 3).

Finally, both pNfL and cNfL levels increased according to the accuracy level of the categories of the Revised El Escorial diagnostic criteria (Brooks et al., 2000) (for pNfL: probable laboratory-supported vs. definite ALS, *p* = 0.001; probable laboratory-supported vs. probable ALS, *p* = 0.002; for cNfL: possible ALS vs. probable ALS, *p* = 0.005; probable laboratory-supported ALS vs. definite ALS, *p* = 0.043; possible ALS vs. definite ALS, *p* = 0.004, Table 3), likely reflecting the effect of the progressive spreading of the neurodegeneration and the increase of body regions involved during the disease course.

### Association Between Cerebrospinal Fluid Neurofilament Light Chain, Plasma Neurofilament Light Chain and the Extent of Upper Motor Neurons and/or Lower Motor Neurons Degeneration

Both cNfL and pNfL were associated with the number of body regions displaying UMN signs (rho = 0.325, *p* < 0.0001; rho = 0.308, *p* = 0.001). Accordingly, both cNfL and pNfL levels significantly raised with increasing number of regions affected by UMN signs only (*p* = 0.008 and *p* = 0.001) or displaying both UMN and LMN signs (*p* = 0.001 and *p* = 0.002). Both results remained statistically significant after adjusting for covariates (i.e., age at sampling, sex, genetic status, basal ALSFRS-R, DPR, MRC, and King’s scores) (cNfL vs. UMN, three regions vs. zero or one region: β = 0.834, CI 0.316–1.636, *p* = 0.042; pNfL vs. UMN, three regions vs. zero or one region: β = 0.609, CI 0.348–1.185, *p* = 0.038; cNfL vs. UMN + LMN, three regions vs. zero or one region: β = 1.003, CI 0.265–1.741, *p* = 0.008; pNfL vs. UMN + LMN, three regions vs. zero or one region: β = 0.529, CI 0.206–1.038, *p* = 0.042).

In contrast, there was no association with the number of LMN affected regions (*p* = 0.467 and *p* = 0.537) (Table 4).

**TABLE 4:** pNfL and cNfL levels according to the extent of UMN and/or LMN degeneration.

		N	pNfL Median (IQR)	N	cNfL Median (IQR)
UMN and LMN degeneration	Zero regions	15	51.8 (32.8–103.9)	10	4161 (2165–8816)
	One region	52	58.2 (32.7–95)	35	4938 (2926–7964)
	Two regions	65	76.8 (48–120.8)	45	7187 (4209–14901)
	Three regions	36	104.0 (64.5–139.1)	23	11052 (6970–15995)
UMN degeneration	Zero regions	10	40.9 (24.3–108.7)	7	3574 (1103–8778)
	One region	29	49.7 (35.8–72)	21	4938 (3814–6590)
	Two regions	60	67.8 (43.4–112.3)	40	5943 (3211–13758)
	Three regions	69	97.2 (58.83–136.9)	45	9440 (5624–14653)
LMN degeneration	Zero regions	6	59.0 (46.9–76.6)	3	4747 (–)
	One region	20	80.4 (22.8–116.0)	11	6263 (881.6–13732)
	Two regions	65	63.4 (38.8–117.3)	41	5784 (3392–13805)
	Three regions	77	79.2 (51.2–112.4)	58	7378 (4496–12147)

*Biomarker values are expressed in pg/ml. Key: ALS, amyotrophic lateral sclerosis; cNfL, cerebrospinal neurofilament light chain; LMN, lower motor neuron; IQR, interquartile range; N, number; pNfL, plasma neurofilament light chain; UMN, upper motor neuron.*

### Prognostic Value of Cerebrospinal Fluid Neurofilament Light Chain and Plasma Neurofilament Light Chain in Amyotrophic Lateral Sclerosis

**FIGURE 2:**
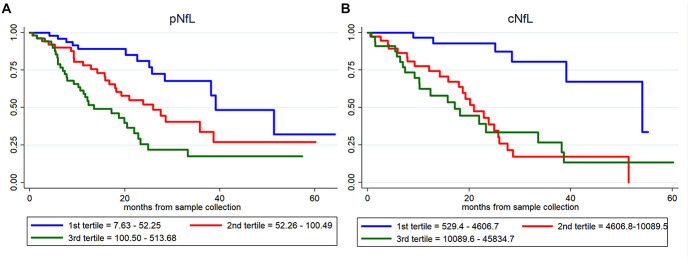
Prognostic value of pNfL and cNfL. Survival curves in ALS patients according to the values of pNfL **(A)** and cNfL **(B)**. A greater increase in baseline cNfL and pNfL levels is associated with shorter survival in patients with ALS. Key: c-NfL, cerebrospinal fluid neurofilament light chain; pNfL, plasma neurofilament light chain.

Based on univariate Cox regression analysis (171 ALS patients; 72 dead), age at sampling (*p* = 0.034), basal ALSFRS-R (*p* < 0.001), DPR (*p* < 0.001), *C9orf72* status (*p* = 0.031), MRC score (*p* = 0.001), King’s score (*p* < 0.001), FVC (*p* < 0.001), cNfL (*p* < 0.001) and pNfL (*p* < 0.001) were identified as predictors of the mortality in ALS patients (Supplementary Table 2). Multivariate Cox regression confirmed the value of both cNfL (HR 2.44, CI 1.52–3.90, *p* < 0.001) and pNfL (HR 2.06, CI 1.31–3.22, *p* = 0.002) as independent predictors of the mortality in ALS (see Supplementary Table 2 for details). Accordingly, ALS patients with higher baseline cNfL and pNfL levels were associated with shorter survival (highest tertile of cNfL vs. lowest tertile of NfL, HR 4.58, CI 1.57–13.41, *p* = 0.005; highest tertile of pNfL vs. lowest tertile of NfL, HR 2.59, CI 1.20–5.58, *p* = 0.015) (Figure 2).

### Longitudinal Trajectories of Plasma Neurofilament Light Chain During the Follow-Up

**FIGURE 3:**
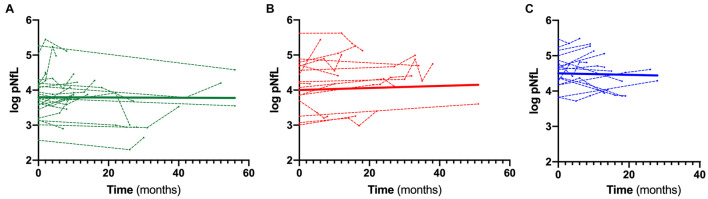
Longitudinal trajectories of pNfL during the follow-up. Overall and single-patient longitudinal pNfL behavior in the slow **(A)**, intermediate **(B)** and fast **(C)** progressors showing a stable longitudinal biomarker trajectory Thick lines represent the overall biomarker trend. Analyses were conducted through a linear mixed effects model. Biomarker values are expressed in the logarithmic scale. Key: pNfL, plasma neurofilament light chain.

When stratifying ALS patients according to the l-DPR, baseline levels of both cNfL and pNfL were significantly higher in ALS fast progressors than the slow progressors (*p* = 0.002 and *p* = 0.001, respectively, Table 5). In contrast, there was no significant rise or decline in the slopes of pNfL levels during follow-up in the three ALS groups (slow β = –0.001, CI –0.009 to 0.007, *p* = 0.773; intermediate β = 0.006, CI –0.002 to 0.013, *p* = 0.126; fast β = –0.0001, CI –0.009 to 0.009, *p* = 0.974, Figure 3), highlighting the overall stability of the biomarker during the disease course.

**TABLE 5:** Longitudinal ALS cohort: patients’ characteristics and biomarkers stratification according to the l-DPR.

Groups (l-DPR)	N	Age at sample mean (SD)	Time from onset to sample (m) mean (SD)	Sex F/M	Type of onset, SPI/BUL/PSE/PYR	cNfL median (IQR)	pNfL median (IQR)
ALS Fast	17	55.6 (13.5)	14.6 (15.6)	8/9	9/6/1/1	9175 (6021–14887)	101.4 (68.7–134.7)
ALS Intermediate	16	67.4 (11.9)	20.8 (10.7)	10/6	11/3/0/2	5520 (3738–8345)	67.8 (43.7–109.9)
ALS Slow	24	64.8 (12.4)	31.7 (24.9)	13/11	17/3/2/2	3250 (2365–5193)	43.4 (31.4–64.4)
*p*-value		0.021*	0.021**	0.71	0.461	0.002°	0.002°°

*Post hoc analysis revealed a significant difference between Fast and Intermediate ALS patients (p = 0.028).

**Post hoc analysis showed a significant difference between Fast and Slow ALS patients (p = 0.021).

*°Post hoc analysis revealed a significant difference between Fast and Slow ALS patients (p = 0.002).*

*°°Post hoc analysis revealed a significant difference between Fast and Slow ALS patients (p = 0.001).*

*The p-values reported directly in the table refer to the multiple-groups comparison analyses. Only the p-values of the comparisons showing a statistically significant difference at the post hoc analysis are further detailed in the table legend.*

*Biomarker values are expressed in pg/ml. Key: ALS, amyotrophic lateral sclerosis; BUL, bulbar; cNfL, cerebrospinal neurofilament light chain; F, females, l-DPR, longitudinal disease progression rate; IQR, interquartile range; m, months; M, Males; N, number; pNfL, plasma neurofilament light chain; PSE, pseudopolyneuritic; PYR, pyramidal; SPI, spinal; SD, standard deviation.*

## Discussion

In the context of motor neuron disease, biofluid markers may aid in the diagnosis of clinically subtle or atypical ALS variants, in the prognostic evaluation of patients and their stratification for clinical trials. Here we confirmed the value of cNfL in distinguishing between patients with ALS and ALS mimics in a large clinical cohort. Additionally, in line with previous studies (Gaiottino et al., 2013; Lu et al., 2015; Benatar et al., 2018; Feneberg et al., 2018; Verde et al., 2019; Ashton et al., 2021), we demonstrated a strong association between cNfL and pNfL, and showed that pNfL also provides a robust diagnostic marker for ALS, especially after excluding patients with peripheral neuropathy, a condition associated with a higher increase of NfL values in plasma than in CSF (Bischof et al., 2018; Mariotto et al., 2018; Sandelius et al., 2018). Given that an extensive clinical and electrophysiological evaluation can reliably identify a PNS involvement, the diagnostic value of pNfL may be considered almost comparable to that of cNfL in the clinical routine. Furthermore, after stratification for age, we found a slight decrease of diagnostic accuracy of pNfL in elderly patients, likely reflecting the physiological increase of the biomarker levels with age, which did not involve the ALS patients, given the marked abnormal concentrations, but that was evident in the ALS mimics cohort.

To address the still debated issue of the pathophysiology of NfL release according to the involvement of upper and lower motor neurons (Zucchi et al., 2020), we investigated the association between biomarker levels and the extent of UMN and LMN degeneration. We found that both pNfL and cNfL levels increased with the number of UMN regions, which is in line with several studies showing a significant correlation between serum (Gille et al., 2019) or CSF (Menke et al., 2015) NfL levels and clinical signs of UMN damage or the extent of corticospinal tract involvement assessed by diffusion tensor MRI (Menke et al., 2015). However, other studies, including our previous evaluation limited to CSF NfL in a smaller cohort, did not confirm this association (Steinacker et al., 2016; Gaiani et al., 2017; Abu-Rumeileh et al., 2020). Beside the possible effects of patient selection and cohort size and the type of assay chosen for the analysis, one likely explanation for these conflicting results relies on the well-known high inter-rater variability in the clinical evaluation of UMN and LMN signs. Indeed, there is still disagreement among neurologists on how to define the presence of UMN-signs given that some consider a preserved reflex in an otherwise atrophic muscle to be a sign of upper motor neuron involvement, while others require the reflex to be hyperactive to reach the same conclusion (Swinnen and Robberecht, 2014). Likewise, given that both clinical and neurophysiological assessment help evaluate LMN involvement, a between-center standardization of neurophysiological techniques is also needed.

In our cohort, both cNfL and pNfL showed higher values in *C9Orf72-*expanded ALS patients than in those with sporadic ALS, likely reflecting the more severe disease course in this patient subgroup. Notably, the current literature does not show full agreement also on this issue with three previous studies supporting our findings (Gendron et al., 2017, Benatar et al., 2020; Huang et al., 2020), and two others not detecting any difference in CSF or serum NFL levels between patients with mutations in *SOD1*, *TARDBP*, *FUS* or the RE of *C9orf72* and sporadic cases (Weydt et al., 2016; Verde et al., 2019).

Another debated issue concerns the potential effect of cognitive impairment on neurofilament levels in ALS. FTD-ALS patients in our cohort presented with higher levels of both pNfL and cNfL than ALS alone, reaching a trend of significance only for the plasma biomarker. Similarly, one study demonstrated higher, although not significant, plasma neurofilament heavy-chain levels in ALS-FTD than in ALS patients (Falzone et al., 2020). However, other studies failed to find a correlation between cognitive functions decline and NfL levels (Gaiani et al., 2017; Feneberg et al., 2018), suggesting that the increase in biomarker levels in ALS is probably relatively independent of the brain regions involved compared to the effect of progression rate. These discordances in the current literature may also be attributable to the small number of ALS-FTD patients enrolled in the available studies. Further studies are, therefore, needed to establish whether the abnormal accumulation of neurofilaments might contribute to the definition of the pathologic ALS-FTD continuum.

On another critical issue, our results confirmed the predictive value on disease progression of cNfL and pNfL assessment (De Schaepdryver et al., 2020). Indeed, our data showed a strong correlation between the biofluid levels of the biomarker and the DPR. Accordingly, when stratifying patients in fast, intermediate, and slow progressors by tertiles, score, biofluid NfL levels were significantly higher in fast progressors compared to the other two groups, in line with previous results (Poesen et al., 2017; Feneberg et al., 2018; Verde et al., 2019; Abu-Rumeileh et al., 2020; Dreger et al., 2021).

In the present study, we also confirmed that both CSF and plasma NfL levels are independent prognostic factors in ALS, even after adjusting for potential clinical prognostic predictors, such as basal ALSFRS-R, genetic status, DPR, MRC, and King’s scores (Benatar et al., 2020). This implies that NfL assessment in both plasma and CSF allows an early diagnosis of ALS and a better stratification of patients for early recruitment in clinical trials, considering the high clinical variability of this devastating disease. Accordingly, a recent study (Benatar et al., 2020) showed that using the baseline serum NfL level as a pharmacodynamic biomarker instead of the ALSFRS-R slope would yield a significant patient sample size saving in a clinical trial.

While the absolute pNfL values varied between patients in our cohort, they remained largely stable in individual patients over time, consistent with previous observations (Lu et al., 2015; Verde et al., 2019). This finding further confirms the potential clinical utility of plasma NfL as a marker of drug effect, provides that the tested novel therapeutics will result in a significant reduction of NfL levels, as recently proved for nusinersen in pediatric spinal muscular atrophy (Darras et al., 2019; Johannsen et al., 2021).

The present study has some limitations. Although we enrolled a significant number of ALS patients, the well-known high variability of the disease did not allow us to draw definitive conclusions about the effect of ALS clinical variants, FTD status, and ALS gene mutations on plasma and CSF NfL levels. Moreover, our demonstration of NfL concentration stability during the disease course was based on the analysis of a relatively small cohort and on longitudinal blood samples collected at non-standardized time points, suggesting caution in interpreting these results. Another partial limitation concerns the small number of ALS patients with a recent onset of symptoms and the absence of pre-symptomatic subjects carrying mutations in ALS genes. The inclusion of such patients could provide additional information about the behavior of biofluids NfL during the pre-symptomatic and early symptomatic phases of the disease, as already pointed out in recent studies (Benatar et al., 2018). Thus, future studies on larger cohorts are needed to validate our results and better explore the NfL behavior during the entire disease course. In conclusion, the results of the present study confirm and extend the available data indicating that both cNfL and pNfL have excellent diagnostic and prognostic performance for symptomatic patients with ALS and support the use of pNfL as a pharmacodynamic marker in clinical trials. However, despite the positive results, to fully understand the diagnostic potential of biofluid NfL in ALS, it would be important to perform more detailed comparisons between ALS patients and homogeneous larger cohorts of single categories of mimic diseases. Furthermore, more extensive prospective multicentric studies on the longitudinal behavior of neurofilament based on standardized methodologies are needed to further assess the role of NfL as a disease progression marker. Finally, a better understanding of how NfL is released in response to pathology, especially in the early disease stages, would also facilitate the use of NfL in the diagnostic work-up and therapeutic trials in ALS.

## Data Availability Statement

The original contributions presented in the study are included in the article/Supplementary Material, further inquiries can be directed to the corresponding author/s.

## Ethics Statement

The study was approved by the Ethics Committee of “Area Vasta Emilia Centro”. The patients/participants provided their written informed consent to participate in this study.

## Author Contributions

VV, AM, and PP: conceptualization and writing—original draft preparation. PP: writing—review and editing based on the critical revision of all authors and supervision. All authors: methodology, formal analysis, and investigation.

## Conflict of Interest

The authors declare that the research was conducted in the absence of any commercial or financial relationships that could be construed as a potential conflict of interest.

## Publisher’s Note

All claims expressed in this article are solely those of the authors and do not necessarily represent those of their affiliated organizations, or those of the publisher, the editors and the reviewers. Any product that may be evaluated in this article, or claim that may be made by its manufacturer, is not guaranteed or endorsed by the publisher.
