# Supplementary material for: Plasma and CSF Neurofilament Light Chain in Amyotrophic Lateral Sclerosis: A Cross-Sectional and Longitudinal Study
Source: Front Aging Neurosci. 2021 Oct 22;13:753242. doi: 10.3389/fnagi.2021.753242 (PMC8569186; doi:10.3389/fnagi.2021.753242)
Supplement: Supplementary file 1 [file Table_1.docx]

Supplementary Material

**Supplementary Table 1:** ALS patients’ clinical features.

| **ALS patients** | **N** |
| --- | --- |
| **ALS phenotypes** | **171** |
| Classic | 116 |
| Bulbar | 19 |
| Prevalent upper motor neuron + PLS | 20 |
| Prevalent lower motor neuron + flail arm | 16 |
| **Continuous variables** | **Median (IQR)** |
| BMI | 24.2 (22-26.7) |
| Total ECAS scores | 106.8 (19.7-116.6) |
| ALS-specific ECAS scores | 79.6 (73-86.6) |
| ALS non-specific ECAS scores | 27.3 (24.3-30.2) |

Key: ALS, amyotrophic lateral sclerosis; BMI, body mass index; ECAS, Edinburgh Cognitive and Behavioural ALS Screen; IQR, interquartile range; PLS, primary lateral sclerosis.
