# Supplementary material for: Plasma and CSF Neurofilament Light Chain in Amyotrophic Lateral Sclerosis: A Cross-Sectional and Longitudinal Study
Source: Front Aging Neurosci. 2021 Oct 22;13:753242. doi: 10.3389/fnagi.2021.753242 (PMC8569186; doi:10.3389/fnagi.2021.753242)
Supplement: Supplementary file 2 [file Table_2.docx]

Supplementary Material

**Supplementary Table 2.** Univariate and multivariate Cox Regression analyses for NfL

and possible clinical predictors of survival in ALS patients

| **Univariate COX regression** | | | |
| --- | --- | --- | --- |
| **Variable** | | **HR (95% CI)** | **p-value** |
| **Sex** | | 1.297 (0.803-2.097) | 0.288 |
| **Age at sampling – years** | | 1.024 (1.001-1.046) | **0.034** |
| **Time from clinical onset to sample - months** | | 0.990 (0.977-1.003) | 0.146 |
| **King’s score** | | 2.970 (2.024-4.359) | **<0.001** |
| **MRC score** | | 0.632 (0.482-0.828) | **0.001** |
| **BMI – kg/m^2^** | | 0.967 (0.907-1.032) | 0.317 |
| **FVC – %** | | 0.962 (0.950-0.972) | **<0.001** |
| **b-DPR** | | 2.134 (1.689-2.969) | **<0.001** |
| **FTD status** | | 1.649 (0.917-2.967) | 0.095 |
| **cNfL – pg/ml** | | 2.567 (1.744-3.779) | **<0.001** |
| **pNfL – pg/ml** | | 2.745 (1.887-3.992) | **<0.001** |
| **Basal ALSFRS-R** | | 0.936 (0.916-0.957) | **<0.001** |
| **Onset type** | **Spinal** | 1.292 (0.768-2.177) | 0.334 |
|  | **Bulbar** | 0.839 (0.301-2.339) | 0.737 |
|  | **Pyramidal** | 0.484 (0.117-2.002) | 0.317 |
|  | **Pseudopolyneuritic** | - | - |
| **Genetic status** | ***SOD1*** | 0.196 (0.269-1.422) | 0.107 |
|  | ***FUS*** | - | - |
|  | ***TARDBP*** | - | - |
|  | ***C9Orf72*** | 1.948 (1.062-3.574) | **0.031** |
| **Multivariate COX regression** | | | |
| **Variable** |  | **HR (95% CI)** | **p-value** |
| **pNfL – pg/ml** | Continuous value | 2.06 (1.31-3.22) | **0.002** |
|  | Low tertile | Ref | Ref |
|  | Mid tertile | 1.84 (0.91-3.75) | 0.09 |
|  | High tertile | 2.59 (1.20-5.589 | **0.01** |
| **cNfL – pg/ml** | Continuous value | 2.44 (1.52-3.90) | **<0.001** |
|  | Low tertile | Ref | Ref |
|  | Mid tertile | 6.24 (2.25-17.27) | **<0.001** |
|  | High tertile | 4.58 (1.57-13.40) | **0.005** |

Key: b-DPR, basal disease progression rate; BMI, body mass index; cNfL, cerebrospinal fluid neurofilament light chain; CI, confidence interval; FVC, forced vital capacity; FTD, frontotemporal dementia; HR, hazard ratio; MRC, Medical Research Council; PLMN, prevalent lower motor neuron; pNfL, plasma neurofilament light chain; PUMN, prevalent upper motor neuron; ref, reference.
